# A Rare Presentation of Angina and Arrhythmia in Absent Left Main Coronary Artery

**DOI:** 10.7759/cureus.12142

**Published:** 2020-12-18

**Authors:** Shoaib Ashraf, Syeda Hafsah Salman, Nisha Ali, Sarthak Kulshreshtha, Muhammad Saad

**Affiliations:** 1 Internal Medicine, Bronx Care Health System Affiliated with Icahn School of Medicine at Mount Sinai, Bronx, USA; 2 Internal Medicine, Bronx Care Health System, Bronx, USA; 3 Cardiology, Bronx Care Health System Affiliated with Icahn School of Medicine at Mount Sinai, Bronx, USA

**Keywords:** congenital absent left main coronary artery, angina, cardiac arrhythmia, coronary angiography

## Abstract

Coronary artery anomalies (CAAs) are congenital disorders with multiple variations in the number, shape, and location of the Ostia of the coronary arterial system. The congenitally absent left main coronary artery (LMCA) is a rare anomaly that can present with benign or fatal complications ahead in life. Diagnosis and management of CAAs are sometimes challenging in low-risk patients.

We present a unique case report of a 69-year-old Hispanic female who presented to the hospital with exercise-induced arrhythmia and angina symptoms. The patient complained of several episodes of chest pain, dizziness, and palpitations for a duration of two months. Electrocardiogram (EKG) and nuclear stress tests were equivocal. The angiogram revealed the separate origin of the left anterior descending artery (LAD) and left circumflex coronary artery (LCX) from the left coronary sinus. This anomaly should be considered in differentials when evaluating patients with angina symptoms.

Congenital absence of LMCA is a rare condition that remains asymptomatic in the majority of the cases. It can present with exertional chest pain, palpitations, syncope, and sudden cardiac death (SCD). Occurrences of angina and arrhythmia should be carefully evaluated, and symptoms should be followed up closely. A coronary angiogram and electrophysiological testing can assist in the diagnosis.

## Introduction

Coronary artery anomalies (CAAs) are congenital disorders characterized by multiple variations in the number, shape, and location of the Ostia of the coronary arteries. CAAs can be benign or life-threatening. It can present with a wide variety of symptoms such as chest pain, myocardial infarction, arrhythmias, sudden death, syncope, and congestive heart failure. Early detection of such an anomaly is of great significance as it can help to prevent serious complications. This article aims to highlight the rare causes of angina and arrhythmia. Our patient was an unusual case of congenital absence of the left main coronary artery (LMCA) who presented with angina symptoms (Video 1).

## Case presentation

**Figure 1:**
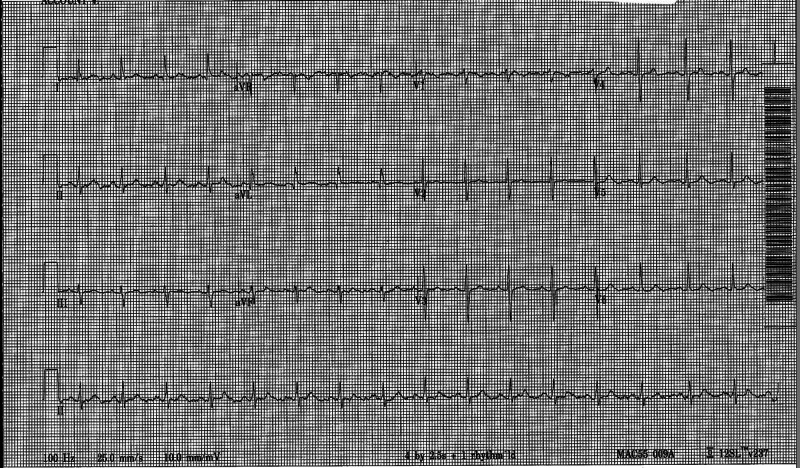
Electrocardiography of the case The 12-lead electrocardiography showed a normal sinus rhythm with a heart rate of 99 beats per minute

A 69-year-old Hispanic woman with a past medical history of hypertension, dyslipidemia, and celiac disease was admitted to the hospital from the cardiology clinic after experiencing exercise-induced angina symptoms. Her primary care physician had referred her to the cardiology clinic on account of two months' history of several episodes of chest pain, dizziness, and palpitations. Chest pain was intermittent, non-radiating, and was associated with exertion at times. She denied syncope, shortness of breath, nocturnal dyspnea, and heartburn. Her medications included aspirin, atorvastatin, carvedilol, losartan, and omeprazole. She had a family history of leukemia, coronary artery disease (CAD), hypertension, and dyslipidemia. She denied smoking, alcohol, and the use of illicit drugs. She was scheduled for a pharmacological nuclear stress test to evaluate for CAD. During the pharmacological nuclear stress test, she developed angina and palpitations. The nuclear stress test was equivocal. Vital signs showed a heart rate of 160 beats per minute (bpm), oxygen saturation of 98% on ambient air, and blood pressure of 136/82 mmHg. The patient received 200 mg IV aminophylline for a possible regadenoson adverse reaction. On physical examination, she was ill-appearing and was in moderate distress. The respiratory and neurologic exams were unremarkable. Her 12-lead electrocardiography (EKG) showed sinus arrhythmia with a heart rate of 99 bpm. The PR interval and QTc measured 142 ms and 441 ms, respectively (Figure 1).

**Figure 2:**
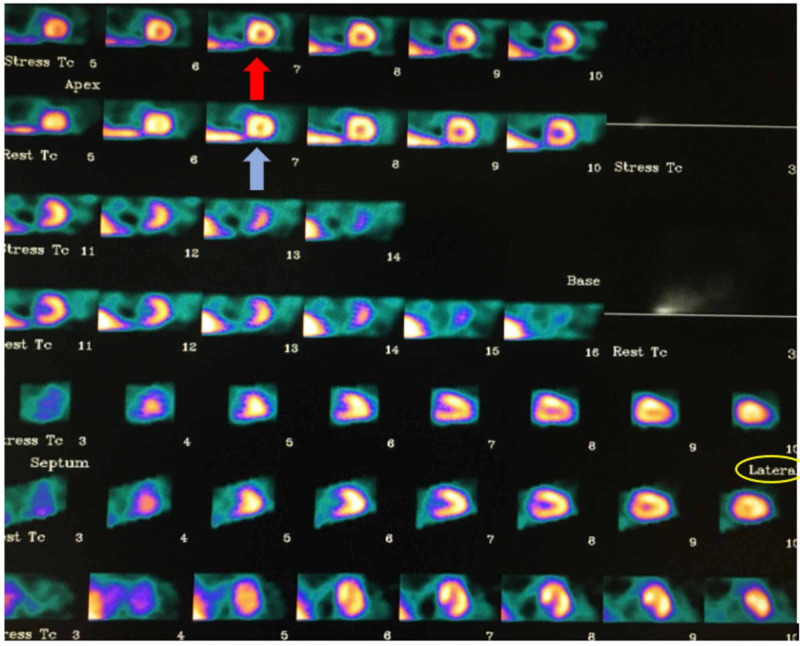
Nuclear stress test images of the case The nuclear stress test showed normal myocardial perfusion in short axis, vertical long-axis, and horizontal long-axis views in both rest and stress images. The red arrow indicates normal perfusion in the stress phase. The blue arrow indicates normal perfusion in the rest phase. The yellow circle shows the lateral view of the nuclear stress test

Laboratory investigations initially showed elevated high-sensitivity troponins of 33 ng/ml and a repeat value of 29 ng/ml with a delta of -4 ng/ml on serial measurements (normal limit: <12 ng/ml). The patient had normal myocardial perfusion images on the nuclear stress test (Figure 2), but because of equivocal EKG changes, the patient underwent cardiac catheterization. The coronary angiogram revealed no significant atherosclerotic lesion or fixed stenosis (Video 1, Video 2), but the LMCA was absent.

**Video 1:** Left heart catheterization - view 1 Left anterior oblique (LAO) with caudal views showing absent left main coronary artery. The left circumflex artery shown here is patent. It is arising from the aorta directly

**Video 2:** Left heart catheterization - view 2 Right anterior oblique (RAO) cranial view of left heart catheterization showing absent left main and patent LAD and LCX LAD: left anterior descending artery; LCX: left circumflex artery

The angiogram revealed the separate origin of the left anterior descending artery (LAD) and left circumflex coronary artery (LCX) from the left coronary sinus (Video 1, Video 2). The patient was diagnosed with a congenital absence of LMCA. Transthoracic echocardiogram prior to admission revealed a left ventricular ejection fraction (LVEF) of 73.5%, estimated right ventricular systolic pressure of 40.69 mmHg, and grade 1 diastolic dysfunction (Video 3, Video 4). The patient did not undergo a coronary flow reserve test by echocardiography due to low suspicion of coronary anomalies. The patient had undergone Holter monitor EKG testing a few years prior to this presentation due to a similar episode of palpitations and chest pain, but the results were not available.

**Video 3:** Transthoracic echocardiogram - view 1 Apical four-chamber view of transthoracic echocardiogram showing normal wall motion and contractility

**Video 4:** Transthoracic echocardiogram - view 2 Apical two-chamber view of transthoracic echocardiogram showing normal anterior and inferior wall contractility

Medical management was primarily considered with conventional anti-angina and anti-hypertensive medications with risk factor modification. An event monitor was provided to rule out any abnormal electrical activity or electrical pathways that can lead to sudden cardiac death (SCD). The patient was discharged two days after admission.

## Discussion

The natural anatomy of coronary arteries commences from the aorta and its course to converge towards the cardiac apex. Generally, it consists of two primary branches, which include the right coronary artery (RCA) and the left coronary artery (LCA). The LCA immediately bifurcates into LAD and LCX, which supply the major areas of the left ventricle and the interventricular septum [1-3]. CAA is the aberrant origination of the coronary artery and/or its branches as opposed to the natural course. It is a rarely reported condition, observed in 0.6%-1.3% of patients in routine angiographic series and 1% of routine autopsies [3]. Coronary anomalies may include anomalies in its origin (coronary aneurysm, absent coronary artery), intramural coronary artery, subendocardial coronary course, split RCA, hypoplastic coronary artery, anomalies of coronary termination, and anastomotic vessels. The absence of LMCA is the most common CAA, with an incidence of approximately 0.5% [2]. In absent LMCA, LAD and LCX originate separately through the aortic sinus. Another commonly found anatomic variant is the origin of LCX from RCA instead of LMCA, with a prevalence of 0.37% [2,4]. It has a predilection for males than females [2]. Embryonically, anomalies in the ectopic growth of the coronary sinus due to defects in the signaling pathway are responsible for the absence of LMCA. It is considered a benign condition with the normal distribution of vessels and no clinical or hemodynamic compromise. The risk of SCD is considered rare in an incidentally discovered coronary anomaly, but it still remains unknown. The risk of SCD is considerably higher when LCX or LAD passes through the aorta and pulmonary artery. However, anomalies of great vessels of the heart have been observed when LCMA originates through the posterior sinus of Valsalva [5,6].

In an asymptomatic or quiescent state, it is usually detected incidentally by CT or invasive coronary angiography [7,8]. Although echocardiography has been proposed as a modality to detect coronary anomalies in severe cases, it is not utilized as a diagnostic modality due to very limited evaluation [8]. It is imperative to estimate and outline the coronary artery origin, distribution, and course whenever CAA is suspected. The standard practice is the use of coronary angiogram, but it is considered limited owing to its two-dimensional imaging and invasive nature [9,10]. Although coronary CT is considered a diagnostic modality to visualize coronary anatomy, the use of radiation and contrast makes its use limited, especially in women of childbearing age. CMR can be utilized safely in this population, but due to its limitation in evaluating small coronary branches, a substantial number of cases can be missed [11,12].

In our case, the patient developed angina symptoms in the post-stress period as a part of stress testing along with the run of non-sustained ventricular tachycardia. Her stress test was equivocal. This event may raise a suspicion for occlusive CAD, anatomic variation in coronary arteries, structural variation such as myocardial bridging, and microvascular angina. The coronary angiography revealed patent coronaries with absent LMCA and the origin of LAD and LCX from the left coronary sinus. Our case is unique because the patient presented with angina and tachyarrhythmia and was found to have an absent LMCA. In contrast, an absent LMCA is reported to have a benign clinical course, and most patients remain asymptomatic. Although we excluded myocardial bridging and acute coronary syndrome (ACS), microvascular ischemia could not be completely ruled out. Published data shows that there might be an association between an anomalous coronary artery and an embryonic defect at the cellular level leading to microvascular ischemia [13]. Upon review of the literature, it was found that absent LMCA can present with angina symptoms, but its presentation with syncope and arrhythmia is a rare phenomenon [14-17]. There are no standardized guidelines regarding the management of this condition [18]. However, caution should be taken during coronary angiography to avoid the misdiagnosis of coronary artery atresia in the presence of separate Ostia of LAD and LCX.

## Conclusions

Congenital absence of LMCA is a rare condition that remains asymptomatic in the majority of the cases. It can present with exertional chest pain, palpitations, syncope, and SCD. Occurrences of angina and arrhythmia should be carefully evaluated, and symptoms should be followed up closely. A coronary angiogram and electrophysiological testing can assist in the diagnosis of coronary anomalies.
